# Guidelines for Neuroprognostication in Critically Ill Adults with Moderate–Severe Traumatic Brain Injury

**DOI:** 10.1007/s12028-023-01902-2

**Published:** 2024-02-17

**Authors:** Susanne Muehlschlegel, Venkatakrishna Rajajee, Katja E. Wartenberg, Sheila A. Alexander, Katharina M. Busl, Claire J. Creutzfeldt, Gabriel V. Fontaine, Sara E. Hocker, David Y. Hwang, Keri S. Kim, Dominik Madzar, Dea Mahanes, Shraddha Mainali, Juergen Meixensberger, Oliver W. Sakowitz, Panayiotis N. Varelas, Christian Weimar, Thomas Westermaier

**Affiliations:** 1 Departments of Neurology and Anesthesiology/Critical Care Medicine, Johns Hopkins University School of Medicine, Baltimore, MD USA; 2 Departments of Neurology and Neurosurgery, University of Michigan, Ann Arbor, MI USA; 3 Department of Neurology, University of Leipzig, Leipzig, Germany; 4 School of Nursing, University of Pittsburgh, Pittsburgh, PA USA; 5 Departments of Neurology and Neurosurgery, University of Florida College of Medicine, Gainesville, FL USA; 6 Department of Neurology, University of Washington, Seattle, WA USA; 7 Departments of Pharmacy and Neurosciences, Intermountain Health, Salt Lake City, UT USA; 8 Department of Neurology, Saint Luke’s Health System, Kansas City, MO USA; 9 Department of Neurology, University of North Carolina at Chapel Hill, Chapel Hill, NC USA; 10 Department of Pharmacy Practice, University of Illinois at Chicago, Chicago, IL USA; 11 Department of Neurology, University of Erlangen-Nuremberg, Erlangen, Germany; 12 Departments of Neurology and Neurosurgery, University of Virginia Health, Charlottesville, VA USA; 13 Department of Neurology, Virginia Commonwealth University, Richmond, VA USA; 14 Department of Neurosurgery, University of Leipzig, Leipzig, Germany; 15 Department of Neurosurgery, Neurosurgery Center Ludwigsburg-Heilbronn, Ludwigsburg, Germany; 16 Department of Neurology, Albany Medical College, Albany, NY USA; 17 Institute of Medical Informatics, Biometry, and Epidemiology, University Hospital Essen, Essen, Germany; 18 BDH-Klinik Elzach, Elzach, Germany; 19 Department of Neurosurgery, Helios Amper Klinikum Dachau, Dachau, Germany; 20 Faculty of Medicine, University of Würzburg, Würzburg, Germany

**Keywords:** Traumatic brain injury, Neurocritical care, Prognosis, Prognostication, Outcome

## Abstract

**Background:**

Moderate–severe traumatic brain injury (msTBI) carries high morbidity and mortality worldwide. Accurate neuroprognostication is essential in guiding clinical decisions, including patient triage and transition to comfort measures. Here we provide recommendations regarding the reliability of major clinical predictors and prediction models commonly used in msTBI neuroprognostication, guiding clinicians in counseling surrogate decision-makers.

**Methods:**

Using the Grading of Recommendations Assessment, Development, and Evaluation (GRADE) methodology, we conducted a systematic narrative review of the most clinically relevant predictors and prediction models cited in the literature. The review involved framing specific population/intervention/comparator/outcome/timing/setting (PICOTS) questions and employing stringent full-text screening criteria to examine the literature, focusing on four GRADE criteria: quality of evidence, desirability of outcomes, values and preferences, and resource use. Moreover, good practice recommendations addressing the key principles of neuroprognostication were drafted.

**Results:**

After screening 8125 articles, 41 met our eligibility criteria. Ten clinical variables and nine grading scales were selected. Many articles varied in defining “poor” functional outcomes. For consistency, we treated “poor” as “unfavorable”. Although many clinical variables are associated with poor outcome in msTBI, only the presence of bilateral pupillary nonreactivity on admission, conditional on accurate assessment without confounding from medications or injuries, was deemed moderately reliable for counseling surrogates regarding 6-month functional outcomes or in-hospital mortality. In terms of prediction models, the Corticosteroid Randomization After Significant Head Injury (CRASH)-basic, CRASH-CT (CRASH-basic extended by computed tomography features), International Mission for Prognosis and Analysis of Clinical Trials in TBI (IMPACT)-core, IMPACT-extended, and IMPACT-lab models were recommended as moderately reliable in predicting 14-day to 6-month mortality and functional outcomes at 6 months and beyond. When using “moderately reliable” predictors or prediction models, the clinician must acknowledge “substantial” uncertainty in the prognosis.

**Conclusions:**

These guidelines provide recommendations to clinicians on the formal reliability of individual predictors and prediction models of poor outcome when counseling surrogates of patients with msTBI and suggest broad principles of neuroprognostication.

**Supplementary Information:**

The online version contains supplementary material available at 10.1007/s12028-023-01902-2.

## Introduction

Millions of patients worldwide suffer a traumatic brain injury (TBI) every year. Moderate–severe TBI (msTBI), defined as Glasgow Coma Scale (GCS) score of 3–12 on admission carries the largest burden of morbidity and mortality [1]. In the United States, msTBI contributes to approximately 30% of all injury-related deaths, resulting in the deaths of 61,000 Americans annually [1, 2]. During the first year, survivors of a TBI often transition between multiple post-acute-care facilities, with many enduring long-term disabilities (physically or cognitively or both). However, research has also shown that with highly intense postacute care and rehabilitation, approximately 20% of patients are functionally independent and living at home 1 year after msTBI [3, 4]. Predicting functional outcome or even mortality in patients with msTBI is extremely difficult, partly because of the high pathophysiological heterogeneity of TBI. Patients may suffer cerebral contusions, subdural or epidural hematomas, traumatic subarachnoid hemorrhage (SAH), intraventricular hemorrhage (IVH), or traumatic axonal injury, isolated or in combination. Severity of TBI, for lack of better classification, has long been classified by the GCS score at presentation after resuscitation (mild: GCS score 13–15; moderate: GCS score 9–12; severe: GCS score ≤ 8). In adults, TBI has a bimodal age prevalence, with young adults often suffering TBIs from motor vehicle crashes and older adults suffering TBIs from falls [1, 2].

After msTBI, withdrawal of life-sustaining treatments (WLST) by surrogate decision-makers is the leading cause of death. The vast majority of msTBI-related deaths, roughly 80%, occur in intensive care units (ICUs) after WLST within the first 2 weeks following injury, and in more than 50%, msTBI-related deaths occur even as early as 72 h [5–7]. To survive and reach rehabilitation, patients with msTBI often need airway and artificial nutrition support, with care by others for their most basic needs for the initial weeks to months or even years. Thus, surrogates must make the difficult decision about continuation or WLST while considering the patient’s potential for long-term disability and diminished quality of life [8–10]. The stakes for WLST decisions are very high: there is the potential for a premature decision leading to the death of a patient who may have survived with a good outcome had treatment been continued [11, 12] or, conversely, prolongation of life with severe physical and cognitive dysfunction, which the patient would not have chosen.

WLST after TBI is highly variable, ranging from 45 to 87% in North American trauma centers [5, 13] and from 0 to 96% in European centers [7], but this variability remains largely unexplained. Although patient and family characteristics, including age, TBI severity, race and ethnicity, religiosity, socioeconomic status, and geography [13], may partly explain this variability in WLST, even after adjusting for important confounders, this variability persists [5]. Previous research has suggested that WLST is the single most important predictor of outcomes of patients with TBI, regardless of injury severity or other patient characteristics [11].

Surrogate decision-makers turn to clinicians to provide a prognostication for these patients with msTBI to make a decision to continue or WLST. For this reason, outcome prognostication is of the highest importance to clinicians and surrogate decision-makers, often families, of patients with msTBI [8]. The objective of this guideline is to provide recommendations on the formal reliability of major clinical predictors that are often associated with msTBI neuroprognostication and provide guidance to ICU clinicians and neurosurgeons who are counseling patients with msTBI and surrogate decision-makers to diminish variable and premature prognostication by clinicians.

### Scope, Purpose, and Target Audience

The scope of these Grading of Recommendations Assessment, Development, and Evaluation (GRADE) guidelines is the prognostication of neurological outcome in critically ill adult patients with msTBI who have received standard-of-care treatment. The purpose of these guidelines is to provide evidence-based recommendations on the reliability of predictors of neurological outcome in critically ill adult patients with msTBI to aid clinicians in formulating a prognosis. The target audience consists of clinicians responsible for such counseling.

### How to Use These Guidelines

These guidelines provide recommendations on the reliability of select demographic and clinical variables, as well as prediction models, when counseling families and surrogate of patients with msTBI. We categorized these predictors as reliable, moderately reliable, or not reliable (Table 1). We based this categorization on a GRADE-based assessment of certainty in the body of evidence, as well as effect size (quantification of predictor accuracy) across published studies. While counseling surrogates, clinicians should explicitly acknowledge the uncertainty that is inherent in prognostication. The degree of uncertainty that is conveyed should be tailored to the reliability of the predictors used during prognostication.

**Table 1:** Reliable and moderately reliable predictors

Category of predictor/model	GRADE criteria					Point estimates of accuracy in the body of evidence	Use during counseling of patients or surrogates?	Presence of additional specific reliable or moderately predictors required for use during counseling?	Suggested language during counseling of patients or surrogates	
	Risk of bias	Inconsistency	Imprecision	Indirectness	Quality of evidence (overall)				Likelihood of outcome	Disclaimer of Uncertainty during counseling
Reliable	One downgrade permitted	Downgrade not permitted	Downgrade not permitted	Downgrade not permitted	Moderate or high	High. Prediction models require AUC > 0.8, no evidence of miscalibration in external validation studies	Yes	Preferred but not absolutely required	Very likely	Present but low
Moderately reliable	One downgrade permitted	Downgrade not permitted	One downgrade permitted	One downgrade permitted	Any	High	Yes	Yes	Likely	Substantial
Moderately reliable clinical prediction models	One downgrade permitted	Downgrade not permitted	One downgrade permitted	One downgrade permitted	Any	High. Prediction models require AUC > 0.7, some miscalibration allowed in external validation studies	Yes	No	Use predicted probability of outcome	The predicted probability is an estimate, subject to considerable uncertainty
Not reliable	Downgrade permitted	Downgrade permitted	Downgrade permitted	Downgrade permitted	Any	Any	No^a^	Not applicable	Not applicable	Not applicable

*AUC* area under the curve, *GRADE* Grading of Recommendations Assessment, Development, and Evaluation

^a^Many predictors designated “not reliable” are practically used by clinicians in formulating and communicating real-world subjective impressions of prognosis. The purpose of these guidelines is to identify predictors, if any, that meet reliable or moderately reliable criteria

A key distinction exists between a “reliable” predictor of outcome in the context of counseling surrogates of patients requiring life-sustaining therapy and an “independent” predictor of outcome. An independent predictor fulfills one criterion: a statistically significant association with the outcome of interest in an appropriately conducted multivariable analysis. In clinical practice, independent predictors of outcome may be used in risk stratification, in selection of patients for targeted treatment (such as chemotherapy regimens for cancer), or as building blocks of clinical prediction models. A reliable predictor in the context of counseling surrogates of patients requiring life-sustaining therapy must be independent but must also fulfill other criteria as depicted in Table 1 and described in the Evidence to recommendation criteria section. Confidence in the accuracy of the predictor should be sufficiently high to overcome concerns about the undesirable consequence of inappropriate WLST.

Reliable predictors, for the purposes of these guidelines, may be used to formulate a prognosis when the appropriate clinical context is present in the absence of potential confounders. These are predictors with clear, actionable thresholds or clinical/radiographic definitions and a low rate of error in prediction of poor outcomes, with at least moderate certainty in the body of evidence. When the prognosis is formulated based on one or more reliable predictors, the clinician may describe the outcome as “very likely” during counseling. Nevertheless, given the inherent limitations in neuroprognostication research, the clinician must acknowledge the presence of uncertainty in the prognosis during counseling.

Moderately reliable predictors may be used for prognostication only when additional reliable or moderately reliable predictors are present, in addition to the appropriate clinical context. These are also predictors with clear, actionable thresholds or clinical/radiographic definitions and a low rate of error in prediction of poor outcomes but with lower certainty in the body of evidence, frequently because of smaller studies that result in imprecision. When the prognosis is formulated based on multiple moderately reliable predictors, the clinician may describe the outcome as “likely” during counseling but must acknowledge “substantial” uncertainty in the prognosis. Moderately reliable clinical prediction models that generate predicted probabilities of outcomes, in contrast, may be used for prognostication during counseling of patients with msTBI and their surrogates in the absence of other reliable or moderately reliable predictors. However, it is recommended that the clinician describe the predicted probability of the outcome as “an objective estimate only, subject to considerable uncertainty”.

Although the panelists recognize that those predictors that do not meet the criteria to be described as reliable or moderately reliable are often used by clinicians in formulating their subjective impressions of prognosis, they have nevertheless been deemed not reliable for the purposes of these guidelines and cannot be formally recommended for prognostication on their own. Variables deemed not reliable, however, may be a component of reliable or moderately reliable prediction models.

## Methods

An in-depth description of the methodology of the literature search is provided in Supplementary Appendix 1. Ethical guidelines were followed. Internal review board approval was not required because data collection was based on an available literature review.

### Selection of Guideline Questions

Candidate predictors were selected based on clinical relevance and the presence of an appropriate body of literature. Candidate predictors and prediction models were considered “clinically relevant” if, in the subjective opinion of the content experts and guideline chairs, the predictor or components of the prediction models were: (1) accessible to clinicians (universal availability of predictors was not required) and (2) likely to be considered by clinicians while formulating a neurological prognosis for patients after msTBI.

An appropriate body of literature was considered present for any predictor evaluated in published studies that included (1) a minimum of 100 study participants and (2) an appropriate multivariate analysis with the predictor of interest, patient’s age, and GCS score or motor GCS score as variables, which are proven and universally accepted parameters associated with the outcome after msTBI. For biomarkers, an appropriate body of literature was considered present if at least one external validation study in addition to the initial report on discovery of the biomarker as an independent predictor was found. For clinical prediction models, an appropriate body of literature was considered present if at least one external validation study in addition to the initial report on development of the model was found.

Based on these criteria, the following candidate predictors were selected:

Clinical variables:

1. Age
2. Pupillary reactivity on admission
3. Admission GCS score or motor GCS subscore following adequate resuscitation
4. Hypotension (systolic blood pressure [SBP] < 90 mm Hg preadmission or in the emergency department)
5. Hypoxia (oxygen saturation < 90% or arterial partial pressure of oxygen (PaO_2_) < 110 mm Hg before or after admission)
6. Major extracranial injury
7. Alcohol intoxication
8. Hypernatremia
9. Prolonged elevated intracranial pressure (ICP) > 20 mm Hg for > 60 min/day
10. Acute kidney injury during ICU treatment
11. Posttraumatic cerebral infarction

Of note, no biomarker was included because none met our prespecified requirements for body of literature as described.

Clinical prediction models:

1. Corticosteroid Randomization After Significant Head Injury (CRASH)-basic model
2. CRASH-CT (CRASH-basic model extended by computed tomography features) model
3. International Mission for Prognosis and Analysis of Clinical Trials in TBI (IMPACT)-core model
4. IMPACT-extended model (core + CT)
5. IMPACT-lab model (core + CT + lab)
6. Marshall CT classification
7. Rotterdam CT
8. Helsinki CT
9. Stockholm CT

The population/intervention/comparator/outcome/timing/setting (PICOTS) question was then framed for the specific candidate predictors as follows: “When counseling family members and/or surrogates of patients with msTBI admitted to an ICU, should [predictor or prediction model, with time of assessment if appropriate] be considered a reliable predictor of [outcome]?”.

### Selection of Outcomes

The outcomes considered “critical” for the systematic review and subsequent formulation of recommendations were functional outcome (average rating 9) assessed at or beyond 6 months after msTBI, mortality (average rating 8) assessed at or beyond discharge, quality of life (average rating 8) assessed at or beyond 6 months after msTBI, and cognitive outcome (average rating 7.67) assessed at or beyond 6 months after msTBI. However, no studies that included quality of life or cognitive outcomes met the full-text screening criteria for the systematic review and could therefore not be included in this guideline. Most articles assessing functional outcome reported “poor” outcome with highly variable definitions (e.g., mostly Glasgow Outcome Scale or Glasgow Outcome Scale Extended with variable dichotomizations or ordinal use, disability-free outcome, Functional Independence Measure, Ranchos Los Amigos Score and other). We list “poor” as equivalent to “unfavorable” for consistency throughout the text of the article. Our Supplementary Table 1 and 2 display the different outcome definitions used in the included articles to represent their variability.

### Systematic Review Methodology

Databases searched included MEDLINE via PubMed, EMBASE, Web of Science, and the Cochrane Database of Systematic Reviews. The librarian search string used for this systematic review is listed in the Supplementary Appendix 1. Full-text screening was performed with the following exclusion criteria: (1) sample size less than 100, (2) studies focused on a highly selected subgroup (such as penetrating TBI or patients with isolated subdural hematoma), (3) studies of predictors not evaluated in multivariate analysis, (4) studies focused on a genetic polymorphism as a predictor, and (5) studies of clinical prediction models that did not report model discrimination. Studies of laboratory biomarkers were included only if the biomarker was considered clinically relevant and had been evaluated in two or more published studies that met other criteria. Studies were screened for several sources of bias while selecting full-text articles for further review.

**Fig. 1:**
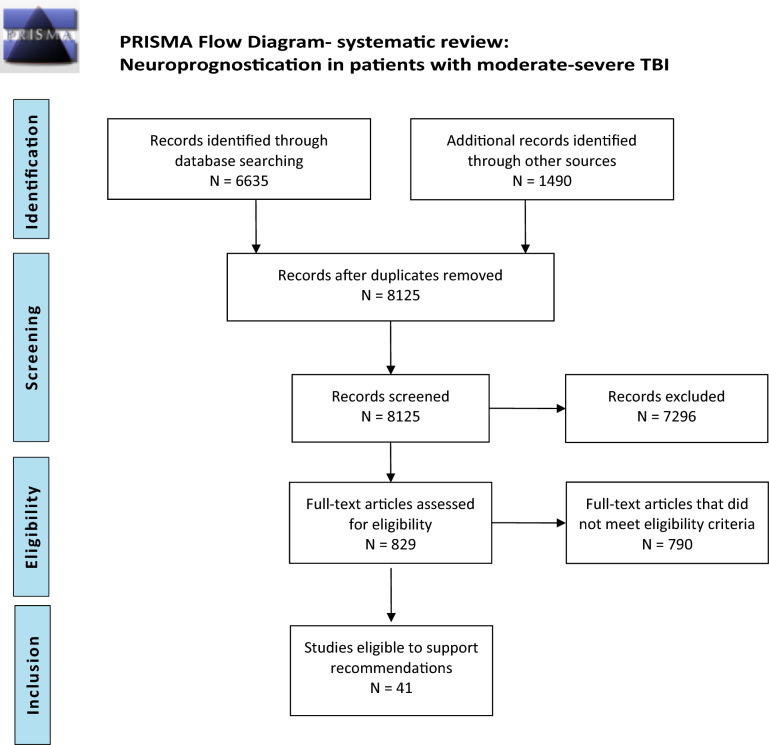
Preferred Reporting Items for Systematic reviews and Meta-Analyses (PRISMA) flow diagram depicting the method of systematic review

Because the librarian’s search was conducted in April 2019, we subsequently performed an additional literature search to include studies until October 2022, using the search words “traumatic brain injury,” “outcome,” and “2019” or “2020” or “2021” or “2022”, respectively. Altogether, 1,490 abstracts were found meeting these criteria. Using the same selection criteria, abstract screening and full-text evaluation was done, resulting in additional 27 articles meeting the GRADE criteria. A total of 8125 abstracts were screened, with 831 full-text articles assessed for eligibility and a total of 41studies included in our qualitative synthesis (Fig. 1).

A summary of individual studies is provided in Supplementary Table 2 (individual predictors) and Supplementary Table 3 (prediction models). A color-coded overview summary of the quality of evidence for individual predictors and prediction models are provided in Table 2. The GRADE evidence profile and summary of findings table for predictors of mortality and functional outcome are shown in Table 3 (individual predictors) and Table 4 (prediction models).

**Table 2:**
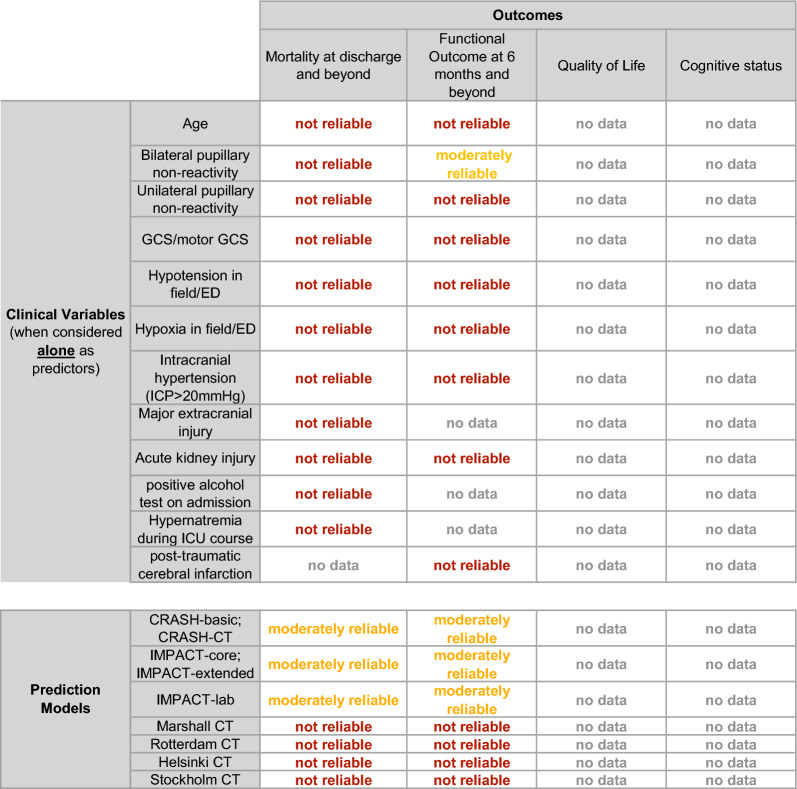
TBI summary table of recommendations

*ED* emergency department, *GCS* Glasgow Coma Scale score, *ICP* intracranial pressure, *ICU* intensive care unit)

**Table 3:** GRADE evidence profile/summary of findings table: neuroprognostication: msTBI

Disease	Outcome	Predictor or model	RoB	Inconsistency	Indirectness	Imprecision	QoE: summary (high/moderate/low/very low)	Summary of findings (narrative of effect size)
Individual predictors								
TBI	Mortality	Age	↓ (self-fulfilling prophecy, statistical analysis, confounding, study attrition, participation)			↓	Low	In hospital mortality: OR per 10 years increase in age 1.47 (95% CI 1.34–1.63); for age > 70, OR 7.72 (1.28–46.7); National Trauma Bank study: age 45–64 aRR 1.97 (95% CI 1.92–2.02); age 65–79 aRR 3.78 (95% CI 3.69–3.86) age > 80 aRR 4.66 (95%CI 4.55–4.76); Age > 70, OR 7.72 (95% CI 1.28–46.67); HR 1.23 (*p* < 0.0001); 6-month mortality: OR IMPACT model 2.4 (95% CI 2.2–2.5) basic model, 2.2 (95% CI 2.0–2.3) extended model, 1.9 (95% CI 1.7–2.1) lab model; correlation *R* = − 0.301, *p* < 0.0001; in combination with GCS and pupil, R2 for linear age increase 26.7
TBI	Mortality	Bilateral pupillary non-reactivity on admission	↓ (self-fulfilling prophecy, participation in one study, study attrition)			↓	Low	In hospital mortality: one or both absent: HR 1.31 (*p* = 0.028) 6-month mortality: IMPACT core: OR 14.2 (95% CI 5.7–35.4); in IMPACT extended: none OR 5.8 (95% CI 1.8–18.5); in combination with age, GCS and pupillary reactivity, R2 for linear GCS-P increase 26.7
TBI	Mortality	Unilateral pupillary non-reactivity on admission	↓ (self-fulfilling prophecy, participation in one study, study attrition)			↓	Low	In hospital mortality: one or both absent: HR 1.31 (*p* = 0.028) 6-month mortality: IMPACT core: one pupil OR 0.7 (95% CI 0.26–1.81); in IMPACT extended: one pupil OR 0.67 (95% CI 0.2–2.3); in combination with age, GCS and pupillary reactivity, R2 for linear GCS-P increase 26.7
TBI	Mortality	Admission GCS/motor GCS	↓ (participation bias, study attrition, confounding, statistical analysis, self-fulfilling prophecy)			↓	Low	GCS beta GCS on arrival beta 0.45 (95% CI 1.27–1.95); GCS 9 aRR 1.37 (95% CI 1.34–1.4); HR 0.61 (*p* < 0.0001); in combination with age, GCS and pupillary reactivity, R2 for linear GCS-P increase 26.7
TBI	Mortality	Hypotension (pre-admission or ED; SBP < 90 mmHg)	↓ (participation bias, study attrition, confounding, self-fulfilling prophecy)			↓	Low	Point estimate: aRR 1.1 (1.06–1.14); OR 3.3 (1.34–8.36); HR 1.67 (*p* = 0.0004)
TBI	Mortality	Hypoxia (pre-admission admission or during ICU stay O2 Sat < 90% or paO2 < 110 mmHg	↓ (participation bias, study attrition, prog factor measurement, confounding, self-fulfilling prophecy)			↓	Low	Point estimate for mortality: OR 1.52–3.3 (95% CI 1.03–1.3 to 2.25–8.36); OR survival: OR 0.54 (0.42–0.69); HR 1.44 (*p* = 0.014)
TBI	Mortality	Prolonged ICP > 20 mmHg	↓ (study attrition, progn. factor measurement, outcome measurement, self-fulfilling prophecy)	↓		↓	Very low	OR 1.84 (95% CI 0.95–3.56); if > 60 min total per day of > 20 mmHg: Early (first 2 days) adjusted OR 34.54 (7.36–162.1); Late (day 3 or later): 7.45 (1.39–40.1);
TBI	Mortality	Major extracranial injury	↓ (participation bias, attrition, confounding, self-fulfilling prophecy)				Moderate	In-hospital mortality: ISS 9–15 aRR 2.07 (1.99–2.16); ISS > 16 aRR 2.97 (2.86–3.09) 6-month mortality: pooled adjusted ORs of 1.46 (95% CI, 1.14–1.85) in moderate; 1.18 (95% CI, 1.03–1.55) in severe TBI patients
TBI	Mortality	Acute Kidney Injury during ICU	↓ (Study participation)			↓	Low	In-hospital mortality: HR 2.05 (95% CI 1.51–3.37) 6-month mortality: HR 2.39 (95% CI 1.7–3.3)
TBI	Mortality	ETOH intoxication	↓ (participation bias, study attrition, prog factor measurement, confounding, self-fulfilling prophecy)	↓			Low	Adjusted OR 0.88 (0.8–0.96); BAC < 100MG/DL OR 1.18 (95% CI 0.53–2.59); BAC > 100MG/DL but < 230MG/DL: OR 0.89 (95% CI 0.55–1.46); BAC > 230MG/DL OR 0.58 (95% CI 0.27–1.25)
TBI	Mortality	hypernatremia	↓ (study attrition, small sample size, confounding, self-fulfilling prophecy)	↓		↓	Very low	After adjusting for DDAVP use (as marker of DI): HR 2 (95% CI 0.81–4.84); time-weighted average Na per 10 mmol/L increase, adjusted OR 4.0 (95% CI 2.1–7.5)
TBI	Unfavorable functional outcome	Age	↓ (study attrition, confounding, statistical analysis, self-fulfilling prophecy)				Moderate	6-month OR IMPACT model 2.4 (95% CI 2.2–2.5) basic model, 2.2 (95% CI 2.0–2.3) extended model, 1.9 (95% CI 1.7–2.1) lab model; correlation *R* = − 0.301, *p* < 0.0001; OR per 10 years of age 1.47 (95% CI 1.34–1.63) for death and 1.49 (95% CI 1.43–1.56) for unfavorable outcome; in combination with GCS and pupillary reactivity, R2 for linear age increase 31.5 for favorable outcome
TBI	Unfavorable functional outcome	Bilateral pupillary non-reactivity on admission	↓ (study attrition, confounding, statistical analysis, self-fulfilling prophecy)				Moderate	6-month IMPACT core: OR 3.3 (95% CI 3.0–3.7); in IMPACT extended: none OR 1.4 (95% CI 1.1–1.7); in IMPACT extended + lab OR 2.1 (95% CI 1.6–2.6); in combination with age, GCS and pupillary reactivity, R2 for linear GCS-P increase 31.5 for favorable outcome
TBI	Unfavorable functional outcome	Unilateral pupillary non-reactivity	↓ (study attrition, confounding, statistical analysis, self-fulfilling prophecy)				Moderate	6-month IMPACT core: OR 1.8 (95% CI 1.6–2.1); in IMPACT extended: OR 1.6 (95% CI 1.4–1.8); in IMPACT extended + lab: OR 1.4 (95% CI 1.1–1.7); in combination with age, GCS and pupillary reactivity, R2 for linear GCS-P increase 31.5 for favorable outcome
TBI	Unfavorable functional outcome	Admission GCS/motor GCS	↓ (study attrition, confounding, statistical analysis, self-fulfilling prophecy)				Moderate	6-month OR mGCS1: 3.9 (3.4–4.5) basic, 3.4 (2.9–4.0) extended, 2.8 (2.1–3.7) lab model; mGCS 2: 5.7 (4.9–6.6) basic, 4.6 (3.9–5.4) extended, 4.3 (3.5–5.4) lab; mGCS 3: 3.0 (2.6–3.5) basic, 2.8 (2.4–3.2) extended, 2.7 (2.2–3.3) lab; mGCS 4: 1.7 (1.5–1.9) basic, 1.6 (1.4–1.8) extended, 1.5 (1.3–1.8) lab; *r* = 0.14, *p* < 0.01; in combination with age, GCS and pupillary reactivity, R2 for linear GCS-P increase 31.5 for favorable outcome
TBI	Unfavorable functional outcome	Hypotension (either SBP < 100 during first 2 weeks in ICU or SBP < 90 mmHg on admission or in ED/field)	↓ (confounding, participation, self-fulfilling prophecy)			↓	Low	6-month OR IMPACT extended: OR 1.8 (1.6–2.1); lab model OR 1.5 (1.2–1.8); single transgression SBP < 90 mmHg < 90% adjusted OR for favorable outcome 1.411 (95% CI 0.1.047–1.9); all transgressions 0.1.249 (95% CI 0.0.858–1.816)
TBI	Unfavorable functional outcome	Hypoxia (prolonged O2 Sat < 90% on admission or during first 2 weeks in ICU)	↓ (confounding, participation, self-fulfilling prophecy)			↓	Low	6-month OR IMPACT extended: OR 1.3 (1.1–1.5); lab model OR 1.4 (1.2–1.7); single transgression O2 Sat < 90% adjusted OR for favorable outcome 1.175 (95% CI 0.868–1.591); all transgressions 0.802 (95% CI 0.566–1.137)
TBI	Unfavorable functional outcome	elevated ICP, > 60 min/day, ICP > 20mmhg;	↓ (participation, study attrition, Progn factor measurement, confounding, self-fulfilling prophecy)			↓	Low	6-month Early IH (day 1,2) 3.99 (1.86–8.54); Late (day 3 and later) IH 2.63 (1.12–6.2)
TBI	Unfavorable functional outcome	Acute Kidney Injury during ICU	↓ (Study participation)			↓	Low	6-month HR 3.04 (95% CI 1.87–4.92)
TBI	Unfavorable functional outcome	Post-traumatic cerebral infarction	↓ (study attrition, self-fulfilling prophecy)			↓	Low	6-month OR GOS 3.88 (95% CI 1.85–8.34)

*AUC* area under the curve, *CI* confidence interval, *DDAVP* desmopressin, *DI* diabetes insipidus, *ETOH* alcohol, *GOS* Glasgow Outcome Scale, *HR* hazard ratio, *IH* intracranial hypertension, *ISS* injury severity score, *OR* odds ratio, *QoE* quality of evidence, *ROB* risk of bias, *RR* relative risk, *SBP* systolic blood pressure, ↓ = quality of evidence downgraded

**Table 4:** GRADE evidence profile/summary of findings table: neuroprognostication: msTBI

Disease	Outcome	Predictor or model	Quality of evidence					Summary of findings (narrative of effect size)
			RoB	Inconsistency	Indirectness	Imprecision	QoE: summary (high/moderate/low/very low)	
Models								
TBI	Mortality	CRASH-basic	↓ ROB: applicability [some mild TBI], self-fulfilling prophecy				Moderate	14-day mortality: AUC 0.8–0.82 (95% CI 0.77–0.75, 0.85–0.87) good calibration; caveat, original model includes mild TBI patients
TBI	Mortality	CRASH CT	↓ ROB: applicability [some mild TBI], self-fulfilling prophecy				Moderate	14-day mortality: AUC 0.83 (0.78–0.87); good calibration; caveat, original model includes mild TBI patients
TBI	Mortality	IMPACT Core	↓ of 7 papers high ROB due to small sample sizes, poor report on methods, variable outcome time points, self-fulfilling prophecy; remaining papers high quality				Moderate	6-month mortality: AUC 0.66–0.9 (95% CI 0.8–0.86, 0.87–0.94), most papers report calibration
TBI	Mortality	IMPACT extended	↓ of 7 papers high ROB due to small sample sizes, poor report on methods, variable outcome time points, self-fulfilling prophecy; remaining papers high quality				Moderate	6-month mortality: AUC 0.71–0.85 (95% CI 0.76–0.81, 0.86–0.89)
TBI	Mortality	IMPACT lab model	↓ two larger paper, two smaller papers, two with high ROB for participation, predictors, outcome, self-fulfilling prophecy)				Moderate	6-month mortality: AUC 0.72–0.80 (95% CI 0.75–0.86)
TBI	Mortality	Marshall CT	↓ high ROB (applicability, lack of calibration, outcome ROB, self-fulfilling prophecy)				Moderate	6-month mortality: AUC 0.635- 0.819 (95% CI 0.769–0.869); calibration reported for one study 12-month mortality: AUC 0.61 (95% CI 0.57–0.65), calibration not reported
TBI	Mortality	Rotterdam CT	↓ 4 papers, three with high ROB for applicability, sample size, analysis, 1/3 mild TBI, self-fulfilling prophecy)		↓		Low	6-month mortality: AUC 0.699–0.875 (95% CI not reported-0.814, not reported-0.936)
TBI	Mortality	Helsinki CT	↓ both papers with high ROB for applicability, analysis, outcome, 1/3 mild TBI, self-fulfilling prophecy lack of calibration)				Moderate	6-month mortality: AUC 0.744 (95% CI not reported); calibration not reported 12- month mortality: AUC 0.74 (95% CI 0.7–0.77); calibration not reported
TBI	Mortality	Stockholm CT	↓ single paper, high ROB (applicability, lack of calibration, outcome, self-fulfilling prophecy ROB)		↓		Low	12- month mortality: AUC 0.77 (95% CI 0.73–0.8); calibration not reported
TBI	Unfavorable functional outcome	CRASH basic	↓ ROB (two of four studies with ROB for participation, applicability, self-fulfilling prophecy calibration not reported)				Moderate	6-month AUC 0.8–0.86 (95% CI 0.75–0.88, 0.84–0.9)
TBI	Unfavorable functional outcome	CRASH CT	↓ ROB (two studies, one with ROB for participation, applicability, self-fulfilling prophecy calibration not reported)				Moderate	6-month AUC 0.86–0.89 95% CI (0.82–0.84, 0.89–0.93)
TBI	Unfavorable functional outcome	IMPACT Core	↓ ROB (1 of 8 studies with ROB for participation, outcome, self-fulfilling prophecy, 2 for analysis, 2 without calibration, 1 with variable outcome time point assessment)				Moderate	6-month AUC 0.71–0.87, 95% CI (0.76–0.81, 0.84–0.91)
TBI	Unfavorable functional outcome	IMPACT extended	↓ ROB (1 of 6 studies with ROB for analysis, self-fulfilling prophecy, no calibration, 1 with variable outcome time point assessment)				Moderate	6-month AUC 0.73–0.88 (95% CI 0.77–0.83, 0.86–0.93)
TBI	Unfavorable functional outcome	IMPACT lab model	↓ ROB (self-fulfilling prophecy, 1 with variable outcome time point assessment, 1 of 4 studies with not enough information to judge ROB)				Moderate	6-month AUC 0.75–0.87 (95% CI 0.82–0.92)
TBI	Unfavorable functional outcome	Marshall CT	↓ ROB (participation [1/3–40% mild TBI], applicability, analysis, self-fulfilling prophecy)		↓		Low	6-month AUC 0.635–0.87 (95% CI 0.82–0.92) 12-month unadjusted AUC 0.54 (95% CI 0.45 – 0.63), calibration not reported
TBI	Unfavorable functional outcome	Rotterdam CT	↓ ROB (participation [1/3–40% mild TBI], applicability, analysis, self-fulfilling prophecy)		↓		Low	6-month AUC 0.682 12-month unadjusted AUC 0.61 (0.53–0.7), calibration not reported
TBI	Unfavorable functional outcome	Helsinki CT	↓ ROB (participation [1/3–40% mild TBI], applicability, analysis, self-fulfilling prophecy)		↓		Low	6-month AUC 0.72–0.75 (95% CI 0.69–0.75) 12-month unadjusted AUC 0.63 (0.54–0.71), calibration not reported
TBI	Unfavorable functional outcome	Stockholm CT	↓ ROB (applicability, lack of calibration, outcome, self-fulfilling prophecy ROB)		↓		Low	12-month AUC 0.7–77 (95% CI 0.61–0.79); calibration not reported

### Evidence to Recommendation Criteria

1. Quality of evidence/certainty in the evidence and effect size: For the purposes of these guidelines, predictors described as “reliable” have both a higher overall certainty in the evidence and greater effect size than “moderately reliable” predictors (Table 1). For reliable predictors, one downgrade was permitted for risk of bias but none for inconsistency, imprecision, or indirectness, and the overall quality of evidence had to be high or moderate. Reliable prediction models were required to demonstrate an area under the receiver operating characteristic curve (AUC) of > 0.8 and no evidence of miscalibration in external validation studies that reported calibration. Downgrades for risk of bias, imprecision and indirectness were permitted for moderately reliable individual predictors, but a downgrade for inconsistency was not. Moderately reliable prediction models were required to demonstrate an AUC > 0.7, and limited miscalibration in some external populations was allowed. Predictors that did not fit “reliable” or “moderately reliable” criteria were classified as “not reliable.”
2. Balance of desirable and undesirable consequences: In the context of msTBI, neuroprognostication is focused on the prediction of poor outcomes in the literature. An accurate prediction of poor outcome is expected to result in grief, a sense of loss, and anxiety about the future. However, a desirable consequence of accurate prediction of a poor outcome is the ability of surrogates and the clinical team to align goals of care to the perceived wishes of the patient with msTBI. Potential benefits to the family and surrogates in this situation include greater certainty and decreased decisional conflict in making patient value-congruent decisions, a sense of closure, and satisfaction from respecting the patient’s wishes. Inaccurate prediction of a poor outcome (i.e., a false-positive prediction of poor outcome), however, may lead to WLST in an individual who would otherwise have made a meaningful recovery. Because WLST almost always leads to death in patients with msTBI, the undesirable consequences of an inaccurate prediction of poor outcome were thought to greatly outweigh the desirable consequences, unless certainty in the evidence of the predictor or prediction model was high (i.e., a low false-positive rate). Other potential undesirable consequences include the risk of events such as loss of airway, hemodynamic instability, inadvertent removal of catheters, and cardiac arrest during transport of a critically ill patient for tests such as brain imaging.
3. Values and preferences: The panel agreed that most individuals, as well as their families and surrogates, would likely consider an inaccurate prediction of poor outcome that led to the death of a patient who might otherwise have had a reasonable recovery to be more undesirable than a prolonged period of uncertainty in the outcome. Therefore, a high certainty in the evidence of predictor or prediction model accuracy was necessary to recommend consideration when counseling families and surrogates on prognosis in this context. The values and preferences of a patient are central to any discussion with surrogates but are also difficult to determine. Shared decision-making based on careful prognostication is critical for the determination of treatment options and goals-of-care decisions.
4. Resource use: Resource use varied across predictors and models. Whereas some predictors, such as the qualitative assessment of the pupillary light response or best motor response, require no significant expenditure of resources, other predictors, such as magnetic resonance imaging (MRI) and measurement of biomarker levels, do involve significant expenditure of resources, for example, in the cost of the diagnostic test itself, the need for personnel to transport the patient and perform the test, and the potential for medical or neurological deterioration from being in the MRI scanner, a less closely monitored environment, for an extended period of time. An accurate prediction of poor outcome, however, may lead to better alignment of goals of care with the patient’s wishes and avoid extended use of resources, over days to years, in patients destined to suffer a poor outcome. The use of resources was therefore thought to favor consideration of a predictor or prediction model during prognostication, contingent on high confidence in its predictive accuracy. In situations in which goals of care have been established and are unlikely to change, however, resource use involved with performance of the test should likely be considered, and expensive tests not expected to alter the treatment plan should be avoided.

### Good Practice Statements

In accordance with recommendations of the GRADE network, these statements were considered by the panel to be actionable, supported by indirect evidence where appropriate, and essential to guide the practice of neuroprognostication. The good clinical practice reflected in these statements lacked a meaningful body of direct supporting evidence (typically because of insufficient clinical equipoise) but was considered by the panel to be unequivocally beneficial.

1. We recommend that prognostication should be performed with consideration of the complete clinical condition and not be based on a single variable (strong recommendation, evidence cannot be graded).
2. We recommend that prognostication should not be performed based only on ultra-early injury characteristics in the first 3 days. TBI is characterized by a primary injury and secondary injuries, including growing hemorrhagic lesions and microcellular processes with possible neurological worsening. In addition, patients with msTBI may have additional nonneurologic trauma (“polytrauma”), which may influence a patient’s hospital course and outcome. Unless irreversible brain stem damage has occurred with imminent death or unless there are documented predetermined wishes by the patient not to receive any critical care support even when the prognosis is uncertain, we suggest a minimum of 3 days or longer of full critical care support, including surgical procedures and, if possible, at least 1–2 weeks of full critical care or medical support before attempting neuroprognostication. Even after 2 weeks, prognosis may be uncertain, especially when patients remain unconscious (strong recommendation, evidence cannot be graded).
3. We recommend that prognostication should be performed carefully with an acknowledgment of uncertainty and without nihilism and should only be performed with consideration of all available evidence and not be based on personal anecdotal experience alone (strong recommendation, evidence cannot be graded).

### Recommendations: Clinical Variables as Predictors

Table 3 (GRADE summary table) shows details on quantitative ranges for the point estimates and 95% confidence intervals (CIs) for each variable and outcome.

### Outcome: Mortality

#### PICOT Question 1

“When counseling family members and/or surrogates of patients with msTBI admitted to an ICU, should age alone be considered a reliable predictor of mortality?”

Description of the predictor: Numeric age assessed and documented at the time of trauma.

Recommendation: When counseling family members and/or surrogates of patients with msTBI admitted to an ICU, we suggest that age alone not be considered a reliable predictor of hospital mortality or disease-related mortality beyond discharge (weak recommendation; low-quality evidence).

Rationale: The body of evidence was downgraded for risk of bias, with various studies demonstrating potential bias in the domains of self-fulfilling prophecy, statistical analysis, study confounding, study attrition, and study participation [14–18]. Furthermore, the evidence was downgraded for imprecision. A large body of literature has assessed and confirmed an independent association of age and poor outcome after msTBI. Consequently, age is also a factor included in more complex prognostic models. However, an appropriate cutoff age has not been identified above which death is certain. Although increasing age was an independent predictor of mortality, with evidence being strongest for patients more than 70 years of age, the criteria for reliability as an individual predictor (compared to a component of a prediction model) were not met, as detailed in the How to use these guidelines and Evidence to recommendation criteria sections. The body of evidence was at high risk of bias from self-fulfilling prophecy. Survival and even good outcome may well be possible in older patients with msTBI.

#### PICOT Question 2

“When counseling family members and/or surrogates of patients with msTBI admitted to an ICU, should bilaterally nonreactive pupils alone, measured on admission, be considered a reliable predictor of mortality?”

Description of the predictor: Bilaterally nonreactive pupils at the time of hospital admission.

Recommendation: When counseling family members and/or surrogates of patients with msTBI admitted to an ICU, we suggest that bilateral pupillary nonreactivity measured on admission be considered a moderately reliable predictor of in-hospital mortality, conditional on accurate assessment without confounding medications or injuries (weak recommendation, low-quality of evidence).

Rationale: Only a few studies met our predetermined criteria to support any recommendations [18–22]. The body of evidence was downgraded for imprecision and risk of bias from participation, study attrition, and the self-fulfilling prophecy because presence of bilaterally fixed pupils may result in early end-of-life decisions [18–22]. Accurate assessment of the pupillary light response is crucial, without confounding by medication, external injury (e.g., orbital trauma), diffuse axonal injury in the mesencephalon, TBI-related seizures, prior surgery, and an overall clinical picture consistent with compression of the third cranial nerve with elevated ICP. Patients may have bilaterally nonreactive pupils but may not meet criteria for brain death and may be kept alive with mechanical ventilation and other life-sustaining measures, including avoidance of WLST, for a prolonged period. Therefore, together with several downgrades in the body of evidence, we suggest that presence of bilaterally nonreactive pupils alone may be considered a moderately reliable predictor of mortality.

#### PICOT Question 3

“When counseling family members and/or surrogates of patients with msTBI admitted to an ICU, should a single nonreactive pupil alone be considered a reliable predictor of mortality?”

Description of the predictor: Single nonreactive pupils at the time of hospital admission.

Recommendation: When counseling family members and/or surrogates of patients with msTBI admitted to an ICU, we suggest that single pupillary nonreactivity alone not be considered a reliable predictor of in-hospital mortality (weak recommendation; low-quality evidence).

Rationale: The body of evidence was downgraded for risk of bias and imprecision [20, 23]. A unilaterally fixed pupil may be the sign of a mass lesion (e.g., herniation from a large subdural hematoma) or traumatic axonal injury at the level of the third nerve nucleus. Thus, many patients with a single reactive pupil may survive (e.g., with time in case of traumatic axonal injury or if rapid surgical intervention is initiated in case of a subdural hematoma). Thus, a unilaterally unreactive pupil measured on admission should not be used as a predictor.

#### PICOT Question 4

“When counseling family members and/or surrogates of patients with msTBI admitted to an ICU, should the GCS score or the motor subscore of the GCS alone following adequate resuscitation be considered a reliable predictor of mortality?”

Description of the predictor: GCS score or motor subscore of the GCS examined at the time of hospital admission following adequate resuscitation.

Recommendation: When counseling family members and/or surrogates of patients with msTBI admitted to an ICU, we suggest that the GCS score or the motor subscore of the GCS alone, assessed on admission after adequate resuscitation, not be considered a reliable predictor of hospital mortality or disease-related mortality beyond discharge (weak recommendation; low-quality evidence).

Rationale: The body of evidence was downgraded for risk of bias, with various studies demonstrating potential bias in the Quality in Prognosis Studies (QUIPS) domains of participation bias, study attrition, confounding, statistical analysis, and self-fulfilling prophecy [14, 15, 18–21]. Inconsistency was present. The GCS score and motor GCS score have been assessed as a single factor or as part of prognostic models. Several studies have shown an association between a low GCS score and mortality. However, the risk for a falsely low GCS score is very high because of an imprecise assessment at the time of assessment in the field and the possible influence of the GCS by other factors (e.g., intoxication, per-intubation medications), resulting in a recommendation for the GCS/motor GCS score not to be a reliable predictor alone for mortality.

#### PICOT Question 5

“When counseling family members and/or surrogates of patients with msTBI admitted to an ICU, should hypotension alone in the field or at the time of hospital admission be considered a reliable predictor of mortality?”

Description of the predictor: Hypotension (SBP < 90 mm Hg) at a single time point or more prior to or at the time of hospital admission.

Recommendation: When counseling family members and/or surrogates of patients with msTBI admitted to an ICU, we suggest that hypotension (SBP < 90 mm Hg) alone, assessed prehospitalization or in the emergency department, not be considered a reliable predictor of hospital mortality or disease-related mortality beyond discharge (weak recommendation; low-quality evidence).

Rationale: The body of evidence was downgraded for risk of bias, with various studies demonstrating potential bias in the domains of participation bias, study attrition, confounding, and self-fulfilling prophecy [14, 20, 24–26]. Inconsistency was present. Blood pressure may not be measured or documented accurately, particularly in the prehospital setting. Furthermore, the thresholds (extent and duration or dose) of hypotension with or without intracranial hypertension that predict death of an individual patient are not clearly defined. Therefore, the false-positive rate is likely to be significant and does not favor considering the predictor alone for prognostication while counseling family and surrogates.

#### PICOT Question 6

“When counseling family members and/or surrogates of patients with msTBI admitted to an ICU, should hypoxia alone in the field, at the time of hospital admission, or during ICU treatment be considered a reliable predictor of mortality?”

Description of the predictor: Hypoxia (oxygen saturation < 90% or PaO_2_ < 110 mm Hg) a single time point or longer prior to or on admission or during the ICU treatment.

Recommendation: When counseling family members and/or surrogates of patients with msTBI admitted to an ICU, we suggest that hypoxia alone, assessed prehospitalization, on admission, or during the ICU treatment (oxygen saturation < 90% or PaO_2_ < 110 mm Hg), not be considered a reliable predictor of hospital mortality or disease-related mortality beyond discharge (weak recommendation; low-quality evidence).

Rationale: The body of evidence was downgraded for risk of bias, with various studies demonstrating potential bias in the QUIPS domains of participation bias, study attrition, prognostic factor measurement, confounding, self-fulfilling prophecy [20, 24, 27, 28], and imprecision. In the studies meeting our prespecified requirements, hypoxia was defined in various ways. Oxygen saturation may not be measured or documented accurately, particularly in the prehospital setting. Furthermore, the thresholds (extent and duration, dose) of hypoxia that predict the death or poor outcome of an individual patient are not clearly defined. Therefore, the false-positive rate is likely to be significant and does not favor considering the predictor alone for prognostication while counseling family and surrogates.

#### PICOT Question 7

“When counseling family members and/or surrogates of patients with msTBI admitted to an ICU, should elevated ICP alone be considered a reliable predictor of mortality?”

Description of the predictor: Elevated ICP (most often defined in the literature as ICP > 20 mm Hg) may be observed at any time during the hospital admission. This could be a single elevated value > 20 mm Hg or a longer-lasting “dose” of ICP > 20 mm Hg (such as duration of > 5 min), both early and in delayed fashion during the hospital course.

Recommendation: When counseling family members and/or surrogates of patients with msTBI admitted to an ICU, we suggest that elevated ICP, including the duration (dose) of early or delayed intracranial hypertension, alone not be considered a reliable predictor of hospital mortality or disease-related mortality beyond discharge (weak recommendation; very low-quality evidence).

Rationale: The body of evidence was downgraded for risk of bias, with various studies demonstrating potential bias in the domains of study attrition, prognostic factor measurement, outcome measurement, and self-fulfilling prophecy [18, 19, 25, 29]. The evidence was further downgraded for imprecision and inconsistency. Although an association of prolonged elevation of ICP with mortality and poor outcome has been documented in a variety of studies, definitions of elevated ICP varied widely, durations were not consistently assessed, and extreme values were not defined. The thresholds (extent and duration, dose) of high ICP that may result in death are uncertain. The false-positive rate may be very high.

#### PICOT Question 8

“When counseling family members and/or surrogates of patients with msTBI admitted to an ICU, should major extracranial injury alone be considered a reliable predictor of mortality?”

Description of the predictor: Many patients with msTBI present as polytrauma patients with other injuries to other organ systems other than the brain. Commonly, the severity of such extracranial injury is measured by the injury severity score.

Recommendation: When counseling family members and/or surrogates of patients with msTBI admitted to an ICU, we suggest that major extracranial injury alone not be considered a reliable predictor for hospital mortality or disease-related mortality beyond discharge (weak recommendation, moderate-quality evidence).

Rationale: The body of evidence was downgraded for risk of bias, with various studies demonstrating potential bias in the domains of study participation, study attrition, study confounding, and self-fulfilling prophecy [14, 20, 30–32]. Extracranial organ trauma may be a separate cause of death or may cause low brain perfusion or oxygenation. However, the spectrum of extracranial injuries additional to msTBI varies widely, with exact definitions for major organ injury or failure completely missing or imprecise in some studies. Thus, major extracranial injury should not be used as a predictor of mortality.

#### PICOT Question 9

“When counseling family members and/or surrogates of patients with msTBI admitted to an ICU, should acute kidney injury (AKI) alone be considered a reliable predictor of mortality?”

Description of the predictor: AKI according to the Kidney Disease Improving Global Outcome criteria during hospitalization is defined as an acute increase in the absolute serum creatinine level of at least 0.3 mg/dL (or an increase of 50%) or a decrease in the urine output (oliguria: urine level < 0.5 mL/kg per hour for more than 6 h).

Recommendation When counseling family members and/or surrogates of patients with msTBI admitted to an ICU, we suggest that AKI during ICU treatment alone not be considered a reliable predictor of hospital mortality or disease-related mortality beyond discharge (weak recommendation; low-quality evidence).

Rationale: The body of evidence was downgraded for risk of bias in the domains of study participation, study attrition, self-fulfilling prophecy, and outcome measurement [33, 34]. The evidence was further downgraded for imprecision. AKI affects 2–12% of patients with msTBI; however, various definitions have been used in different studies. Also, the different stages of AKI were not analyzed for their impact on mortality. Furthermore, AKI may be fully reversible; therefore, the risk for a false-positive finding may be very high. Thus, AKI should not be used as a predictor for mortality.

#### PICOT Question 10

“When counseling family members and/or surrogates of patients with msTBI admitted to an ICU, should the presence of alcohol in the blood alone on admission be considered a reliable predictor of mortality?”

Description of the predictor: Positive alcohol test on admission.

Recommendation: When counseling family members and/or surrogates of patients with msTBI admitted to an ICU, we suggest that positive testing for alcohol on admission alone not be considered a reliable predictor of hospital mortality or disease-related mortality beyond discharge (weak recommendation, low-quality evidence).

Rationale: The body of evidence was downgraded for risk of bias, with various studies demonstrating potential bias in the domains of study participation, study attrition, prognostic factor measurement, study confounding, and self-fulfilling prophecy [35, 36]. Inconsistency was present. Intoxication with alcohol may cause a falsely low GCS score on admission and subsequently a false stratification of patients into msTBI. Intoxicated patients may improve significantly as their alcohol levels diminish with time, often by the next day. Thus, a positive alcohol test on admission should not be used as a predictor for mortality according to the PICOTS question.

#### PICOT Question 11

“When counseling family members and/or surrogates of patients with msTBI admitted to an ICU, should hypernatremia alone be considered a reliable predictor of mortality?”

Description of the predictor: Hypernatremia defined as a serum sodium level > 160 mmol/L during the ICU stay.

Recommendation: When counseling family members and/or surrogates of patients with msTBI admitted to an ICU, we suggest that hypernatremia during ICU treatment alone not be considered a reliable predictor of hospital mortality or disease-related mortality beyond discharge (weak recommendation, very low-quality evidence).

Rationale: The body of evidence was downgraded for risk of bias, with various studies demonstrating potential bias in the domains of study attrition, confounding, and self-fulfilling prophecy [37, 38]. Inconsistency and imprecision were present. From a pathophysiological point of view, hypernatremia may be indicative of a particularly severe TBI with hypothalamic dysfunction or may be a marker of the frequent administration of osmotherapy (mannitol, hypertonic saline). Hypernatremia, especially when iatrogenic, is a treatable and reversible condition. Numerous studies have found an association between the occurrence of hypernatremia and outcome/mortality. However, only very few studies met our criteria. These studies had different cutoff points for hypernatremia. One study suggested that possibly co-occurring hyperchloremia, and not hypernatremia itself, may be associated with mortality [37].

### Outcome: Functional Outcome at 6 Months and Beyond

#### PICOT Question 12

“When counseling family members and/or surrogates of patients with msTBI admitted to an ICU, should age alone be considered a reliable predictor of poor functional outcome at 6 months and beyond?”

Description of the predictor: Numeric age assessed and documented at the time of trauma and correlated with outcome.

Recommendation: When counseling family members and/or surrogates of patients with msTBI admitted to an ICU, we suggest that age alone not be considered a reliable predictor of functional outcome at 6 months and beyond (weak recommendation, moderate-quality evidence).

Rationale: The body of evidence was downgraded for risk of bias, with various studies demonstrating potential bias in the domains of study attrition, confounding, statistical analysis, and self-fulfilling prophecy [15, 16, 23, 39–41]. A large body of literature has assessed and confirmed an independent association of age with poor outcome after msTBI. Age is also a factor included in several prognostic models. In one study, a linear correlation was observed between poor functional outcome and increasing age, with an odds ratio of 1.49 for every 10 years of age [16]. However, the criteria for reliability as an individual predictor (as compared to a component of a prediction model) were not met, as detailed in the How to use the guidelines and Evidence to recommendation criteria sections. The body of evidence was at high risk of bias from the self-fulfilling prophecy. Individual older patients with severe TBI may achieve a good outcome when other clinical variables are favorable. A threshold age beyond which poor outcome is inevitable has not been identified.

#### PICOT Question 13

“When counseling family members and/or surrogates of patients with msTBI admitted to an ICU, should bilaterally nonreactive pupils alone, measured on admission, be considered a reliable predictor of poor functional outcome at 6 months and beyond?”

Description of the predictor: Bilaterally nonreactive pupils at the time of hospital admission.

Recommendation: When counseling family members and/or surrogates of patients with msTBI admitted to an ICU, we suggest that bilateral pupillary nonreactivity alone, measured on admission, is a moderately reliable predictor of poor functional outcome at 6 months and beyond (weak recommendation; moderate-quality evidence).

Rationale: The body of literature assessing the correlation of bilaterally fixed pupils and functional outcome was downgraded for risk of bias from study attrition, confounding, statistical analysis, and the self-fulfilling prophecy [21, 23]. We emphasize that this recommendation is conditional on accurate assessment of the pupillary light response and the consideration of confounding factors, such as medication, external injury (e.g., orbital trauma), diffuse punctual axonal injury in the mesencephalon, TBI-related seizures, prior surgery, or an overall clinical picture consistent with compression of the third cranial nerve with elevated ICP. Conditional on exclusion of factors that may potentially lead to an incorrect assessment, the presence of bilaterally nonreactive pupils was deemed a moderately reliable predictor of poor functional outcome based on the existing literature. However, this variable could not be recommended as a reliable predictor because of several downgrades in the body of evidence as per our definition of a reliable predictor (Table 1).

### PICOT Question 14

“When counseling family members and/or surrogates of patients with msTBI admitted to an ICU, should a unilaterally nonreactive pupil alone be considered a reliable predictor of poor functional outcome at 6 months and beyond?”

Description of the predictor: Unilateral nonreactive pupil at the time of hospital admission.

Recommendation: When counseling family members and/or surrogates of patients with msTBI admitted to an ICU, we suggest that unilateral pupillary nonreactivity alone, measured on admission, not be considered a reliable predictor of poor functional outcome at 6 months and beyond (weak recommendation; moderate-quality evidence).

Rationale: The body of evidence was downgraded for risk of bias in the domains of study attrition, confounding, statistical analysis, and self-fulfilling prophecy [21, 23]. In addition, a unilaterally fixed pupil may be the sign of traumatic axonal injury or a mass lesion (e.g., subdural hematoma), which may be treated by surgical evacuation. Recovery may be quick and complete, and therefore the risk for a false-positive finding may be very high.

#### PICOT Question 15

“When counseling family members and/or surrogates of patients with msTBI admitted to an ICU, should the GCS score (or GCS motor score) alone following adequate resuscitation be considered a reliable predictor of poor functional outcome at 6 months and beyond?”

Description of the predictor: The GCS score or the motor subscore of the GCS examined at the time of hospital admission following adequate resuscitation.

Recommendation: When counseling family members and/or surrogates of patients with msTBI admitted to an ICU, we suggest that the GCS score (or GCS motor score) alone, measured on admission and following adequate resuscitation, not be considered a reliable predictor of poor functional outcome at 6 months and beyond (weak recommendation; moderate-quality evidence).

Rationale: The body of evidence was downgraded for risk of bias, with various studies demonstrating potential bias in the domains of study attrition, confounding, statistical analysis, and self-fulfilling prophecy [15, 21, 23, 40]. Inconsistency was present. The GCS score and GCS motor score have been assessed as a single factor or in the framework of prognostic models, and a correlation between a poor GCS score and mortality and poor functional outcome has been demonstrated. However, the false-positive rate may be very high because of an imprecise assessment at the time of assessment in the field and the possible influence of the GCS assessment by other factors (e.g., intoxication, medications).

#### PICOT Question 16

“When counseling family members and/or surrogates of patients with msTBI admitted to an ICU, should hypotension alone in the field or at the time of hospital admission be considered a reliable predictor of poor functional outcome at 6 months and beyond?”

Description of the predictor: Hypotension (SBP < 90 mm Hg) prior to or at the time of hospital admission.

Recommendation: When counseling family members and/or surrogates of patients with msTBI admitted to an ICU, we suggest that hypotension alone, prior to or at the time of hospital admission, not be considered a reliable predictor of poor functional outcome at 6 months and beyond (weak recommendation; low-quality evidence).

Rationale: The body of evidence was downgraded for risk of bias, with various studies demonstrating potential bias in the domains of confounding, participation, and self-fulfilling prophecy [23–25]. Imprecision was present. The body of literature does not sufficiently address the duration and degree of hypotension, nor does it sufficiently consider the simultaneous presence of elevated ICP. Thus, hypotension should be a target for immediate treatment and should not be used as a predictor of poor outcome for the purpose of counseling patient surrogates.

#### PICOT Question 17

“When counseling family members and/or surrogates of patients with msTBI admitted to an ICU, should hypoxia alone in the field or at the time of hospital admission be considered a reliable predictor of poor functional outcome at 6 months and beyond?”

Description of the predictor: Hypoxia (oxygen saturation < 90% or PaO_2_ < 110 mm Hg) prior to or at the time of hospital admission.

Recommendation: When counseling family members and/or surrogates of patients with msTBI admitted to an ICU, we suggest that hypoxia alone, prior to or at the time of hospital admission, not be considered a reliable predictor of poor functional outcome at 6 months and beyond (weak recommendation; low-quality evidence).

Rationale: The body of evidence was downgraded for risk of bias, with various studies demonstrating potential bias in the domains of confounding, participation, and self-fulfilling prophecy [23–25]. In the studies meeting our criteria, hypoxia was defined in several different ways. In addition, the body of literature does not sufficiently address the duration of hypoxia, nor does it consider other confounders of outcome sufficiently and consistently. Thus, hypoxia should be a target of immediate therapy but not be used as a predictor of poor outcome for the purpose of counseling patient surrogates.

#### PICOT Question 18

“When counseling family members and/or surrogates of patients with msTBI admitted to an ICU, should elevated ICP alone be considered a reliable predictor of poor functional outcome at 6 months and beyond?”

Description of the predictor: Elevated ICP (ICP > 20 mm Hg) during ICU treatment. This could be a single elevated value > 20 mm Hg or a longer-lasting dose of ICP > 20 mm Hg (such as duration of > 5 min), both early and in delayed fashion during the ICU course.

Recommendation: When counseling family members and/or surrogates of patients with msTBI admitted to an ICU, we suggest that elevated ICP alone during the treatment in the ICU not be considered a reliable predictor of poor functional outcome at 6 months and beyond (weak recommendation; low-quality evidence).

Rationale: The body of evidence was downgraded for risk of bias, with various studies demonstrating potential bias in the domains of participation, study attrition, prognostic factor measurement, study confounding, and the self-fulfilling prophecy [19, 25, 40, 42]. Imprecision was present. Definitions of elevated ICP varied in the included studies, duration of ICP elevation was not consistently assessed, and extreme values were not defined or considered. Odds ratios varied between the different studies. Thus, elevated ICP should be a target for immediate treatment and should not be used as a predictor of poor functional outcome for the purpose of counseling patient surrogates.

#### PICOT Question 19

“When counseling family members and/or surrogates of patients with msTBI admitted to an ICU, should AKI alone be considered a reliable predictor of poor functional outcome at 6 months and beyond?”

Description of the predictor: AKI injury according to the Kidney Disease Improving Global Outcome criteria during hospitalization.

Recommendation: When counseling family members and/or surrogates of patients with msTBI admitted to an ICU, we suggest that AKI alone not be considered a reliable predictor of poor functional outcome at 6 months and beyond (weak recommendation; low-quality evidence).

Rationale: AKI is one of several ICU complications that may be associated with outcome. The body of evidence is small and was downgraded for study participation bias [33]. Imprecision was present. The different stages of AKI were not analyzed for their association with functional outcome. Furthermore, AKI may be fully reversible and, hence, the false-positive rate may be very high.

#### PICOT Question 20

“When counseling family members and/or surrogates of patients with msTBI admitted to an ICU, should a posttraumatic cerebral infarction alone be considered a reliable predictor of poor functional outcome at 6 months and beyond?”

Description of the predictor: Ischemic brain infarct appearing on CT and/or MRI not present on admission.

Recommendation: When counseling family members and/or surrogates of patients with msTBI admitted to an ICU, we suggest that posttraumatic cerebral infarction alone not be considered a reliable predictor of poor functional outcome at 6 months and beyond (weak recommendation; low-quality evidence).

Rationale: The body of evidence was small and downgraded for risk of bias, with various studies demonstrating potential bias in the domains of study attrition and self-fulfilling prophecy [17]. Imprecision was present. “Infarction” has been defined as a wedge-like lesion on CT or MRI, and it is not clear how reliably this can be assessed among different providers interpreting the imaging. The presence of infarction may be a sign of herniation, acute thromboembolism (with or without dissection), or small-vessel disease in noneloquent areas. The mechanism of the infarction may determine the association with outcome, but this was not consistently assessed in the literature.

## Recommendations: Clinical Prediction Models

Table 4 (GRADE summary table) shows details on quantitative ranges for the models’ discrimination (AUC) and if and how calibration was determined.

### Outcome: Mortality at Hospital Discharge and Beyond

#### PICOT Question 21

“When counseling family members and/or surrogates of patients with msTBI admitted to an ICU, should the CRASH-basic or CRASH-CT models be considered reliable predictors of mortality?”

Description of the predictor: The CRASH models were developed in a large cohort of international patients with TBI (derivation *n* = 10 008, validation *n* = 8,509 with GCS score of 14) who were enrolled in the CRASH clinical trial from low-, middle-, and high-income countries [43]. The model was developed to predict short-term (14-day) mortality and death and severe disability at 6 months after injury in patients with TBI. The factors included in the CRASH-basic model are age, GCS score, pupillary reactivity, and the presence of major extracranial injury. The CRASH-CT model additionally included the following head CT findings: presence of petechial hemorrhages, obliteration of the third ventricle or basal cisterns, subarachnoid bleeding, midline shift (MLS), and nonevacuated hematoma. Both models have been validated in many different cohorts. Although the models were developed in patients with TBI who were both moderate–severe and mild, the models are very well known in the field and were developed and validated in a large population. An online calculator (including display of 95% CI) is available for use at http://crash2.lshtm.ac.uk/Risk%20calculator/index.html.

Recommendation: When counseling family members and/or surrogates of patients with msTBI admitted to an ICU, we suggest that the CRASH-basic and CRASH-CT clinical prediction models be considered moderately reliable predictors of short-term (14-day) mortality (weak recommendation; moderate-quality evidence).

Rationale: The body of evidence was downgraded for risk of bias for applicability (mild TBI [GCS scores 13 and 14] included in the model derivation) [43]. Risk of bias from the self-fulfilling prophecy was present. Derivation and validation studies did not report when or whether WLST was performed, and it was unclear if the CRASH-basic [43–46] or CRASH-CT models [43, 46] may have been used systematically to inform treatment limitation.

One caveat in the initial derivation and external validation study was that models had to be developed separately in the cohorts of high-income and low-income countries because of interaction of country’s income level and several predictors [43]. However, the external validity of both CRASH models predicting the outcome of patients with msTBI was subsequently confirmed in several studies [44–46].

#### PICOT Question 22

“When counseling family members and/or surrogates of patients with msTBI admitted to an ICU, should the IMPACT-core or IMPACT-extended (core + CT) models be considered reliable predictors of mortality?”

Description of the predictor: The IMPACT models were developed using 8,509 patients with msTBI from a single database that pooled 11 studies (eight randomized controlled trials and three observational studies) conducted between 1984 and 1997 [23]. The IMPACT-core model was developed on the entire cohort and contains the following variables: age, GCS motor subscore, and pupillary reactivity. The IMPACT-extended (core + CT) model was developed using a sample of 6,999 patients and additionally contains the following variables: presence of hypotension (SBP < 90 mm Hg) in the field or emergency department, hypoxia (oxygen saturation < 90%) in the field or emergency department, Marshall CT classification, presence of traumatic SAH, and presence of epidural hemorrhage (both on CT). A free online risk calculator is available, but it does not display 95% CI (http://tbi-impact.org/?p=impact/calc).

Recommendation: When counseling family members and/or surrogates of patients with msTBI admitted to an ICU, we suggest that the IMPACT-core and IMPACT-extended (core + CT) clinical prediction models be considered moderately reliable predictors of 6-month mortality (weak recommendation; moderate-quality evidence).

Rationale: The body of evidence was downgraded for risk of bias in some studies [47, 48] because of small sample sizes, poor reporting of methods, and variable time points of outcome assessment [45, 47, 48]. However, other studies were of high quality, with low risk of bias [23, 44, 46, 49]. All studies did not address the impact of WLST and were therefore at risk of bias from the self-fulfilling prophecy. However, the IMPACT models are the most widely validated models in msTBI, with several contemporary validation studies.

#### PICOT Question 23

“When counseling family members and/or surrogates of patients with msTBI admitted to an ICU, should the IMPACT core + CT + lab model be considered a reliable predictor of mortality?”

Description of the predictor: This model was also developed using the same pooled database as the aforementioned models (IMPACT database) but restricted to a smaller sample size (*n* = 3,554) because of the nonavailability of some laboratory values in the pooled derivation cohort [23]. The IMPACT core + CT + lab model contains the same variables as the IMPACT core + CT model plus glucose and hemoglobin values from admission. A free online risk calculator is available, but it does not display 95% CI (http://tbi-impact.org/?p=impact/calc).

Recommendation: When counseling family members and/or surrogates of patients with msTBI admitted to an ICU, we suggest that the IMPACT core + CT + lab clinical prediction model be considered a moderately reliable predictor of 6-month mortality (weak recommendation; moderate-quality evidence).

Rationale: Overall, risk of bias was present in the following the Prediction Model Risk of Bias Assessment Tool (PROBAST) domains: participation, predictors, and outcomes (variable time point assessment) [48, 50]. All studies did not address the impact of WLST and were therefore at risk of bias from the self-fulfilling prophecy.

#### PICOT Question 24

“When counseling family members and/or surrogates of patients with msTBI admitted to an ICU, should the Marshall CT classification be considered a reliable predictor of mortality?”

Description of the predictor: The Marshall CT classification includes six categories of head CT findings for msTBI with binary presence or absence of any intracranial abnormalities, compression of basal cisterns, MLS > 5 mm, presence of any mass lesion > 25 cm^3^, and planned or performed evacuation of mass lesions [51]. The detailed classification has been described as follows:

1. Diffuse injury I: no visible intracranial pathological finding/injury on head CT
2. Diffuse injury II: some visible intracranial pathological findings with open basal cisterns, MLS 0–5 mm, and/or no mixed/high density lesion > 25 cm.^3^
3. Diffuse injury III: Basal cisterns compressed or absent with MLS of 0–5 mm and no mixed/high density lesion > 25 cm.^3^
4. Diffuse injury IV: MLS > 5 mm but no mixed/high density lesion > 25 cm.^3^
5. Diffuse injury V: any mass lesion surgically evacuated
6. Diffuse injury VI: any mixed/high density mass lesion > 25 cm.^3^

Recommendation: When counseling family members and/or surrogates of patients with msTBI admitted to an ICU, we suggest that the Marshall CT classification not be considered a reliable predictor of mortality assessed at 6 months or beyond (weak recommendation; moderate-quality evidence).

Rationale: The body of evidence was downgraded for risk of bias. Applicability concerns were present because a quarter of patients in the largest study [52] and a third of patients in another study [47] had only mild TBI. Calibration was not consistently assessed. Outcome assessment was not clearly described, or the time point of outcome assessment was highly variable [45]. None of the studies that met our criteria assessed hospital mortality as an outcome. The original Marshall publication [51] did not meet our inclusion criteria because it did not report model discrimination. All studies did not address the impact of WLST and were therefore at risk of bias from the self-fulfilling prophecy.

#### PICOT Question 25

“When counseling family members and/or surrogates of patients with msTBI admitted to an ICU, should the Rotterdam CT score be considered a reliable predictor of mortality?”

Description of the predictor: The Rotterdam CT score was developed to predict mortality at 6 months [53] and is the sum of all subscoring items plus 1. Subscoring items include basal cisterns (0, normal; 1, compressed; 2, absent), MLS (0, no shift or 5 mm; 1, shift > 5 mm), epidural mass lesion (0, present; 1, absent), and intraventricular blood or traumatic SAH (0, present; 1, absent).

Recommendation: When counseling family members and/or surrogates of patients with msTBI admitted to an ICU, we suggest that the Rotterdam CT score not be considered a reliable predictor of 6-month mortality (weak recommendation; low-quality evidence).

Rationale: The body of evidence was downgraded for risk of bias. Applicability concerns were present because about a quarter to a third of patients had only mild TBI [47, 52]. In addition, several studies had a small sample size [45, 47, 52, 53]. Two studies had a sample size > 900 [52, 53]. Calibration was often not reported, and reporting of study analysis was frequently incomplete. All studies did not address the impact of WLST and were therefore at risk of bias from the self-fulfilling prophecy.

#### PICOT Question 26

“When counseling family members and/or surrogates of patients with msTBI admitted to an ICU, should the Helsinki CT score be considered a reliable predictor of mortality?”

Description of the predictor: The Helsinki CT score was developed to predict mortality at 6 months [47] and is the sum score of the following variables: mass lesion type (2, subdural hematoma; 3, intracerebral hemorrhage; − 3 epidural hematoma), mass lesion size (2, hematoma volume > 25 cm^3^), presence of IVH (3, present), and state of suprasellar cisterns (0, normal; 1, compressed; 5, obliterated). The range of the sum score is − 3 (min) to 14 (max).

Recommendation: When counseling family members and/or surrogates of patients with msTBI admitted to an ICU, we suggest that the Helsinki CT score not be considered a reliable predictor of 6-month mortality (weak recommendation; moderate-quality evidence).

Rationale: The body of evidence was downgraded for risk of bias. Applicability concerns were present because about a quarter to a third of patients had only mild TBI [47, 52]. In addition, outcome was assessed at variable time points [52], sample size was often low (only one study had a sample size > 900) [52], and calibration was not consistently reported [47, 52]. All studies did not address the impact of WLST and were therefore at risk of bias from the self-fulfilling prophecy.

#### PICOT Question 27

“When counseling family members and/or surrogates of patients with msTBI admitted to an ICU, should the Stockholm CT score be considered a reliable predictor of mortality?”

Description of the predictor: The Stockholm CT score puts great emphasis on the thickness and presence of SAH and IVH and includes MLS as a continuous variable (not dichotomized) in addition to diffuse axonal injury. The score is calculated by first calculating the traumatic SAH subscore: SAH in convexities (1, if 1–5 mm; 2 if > 5 mm) + SAH in basal cisterns (1, if 1–5 mm; 2 if > 5 mm) + IVH (2 if present) (range 0–6). The final score is then calculated as follows: MLS (mm)/10 + traumatic SAH score/2–1 (if epidural hemorrhage present) + 1 (if diffuse axonal injury present in basal ganglia, splenium, or brainstem) + 1 (if dual-sided subdural hematoma) + 1.

Recommendation: When counseling family members and/or surrogates of patients with msTBI admitted to an ICU, we suggest that the Stockholm CT score not be considered a reliable predictor of 6-month mortality (weak recommendation; low-quality evidence).

Rationale: The body of evidence was downgraded because of risk of bias present in the following PROBAST domains: applicability (nearly one quarter of included patients had only mild TBI), outcome (was assessed at variable time points), and analysis (no calibration reported). All studies did not address the impact of WLST and were therefore at risk of bias from the self-fulfilling prophecy. The body of evidence was also downgraded for indirectness because the body of evidence included a large proportion of patients with mild TBI.

### Outcome: Functional Outcome at 6 Months and Beyond

#### PICOT Question 28

“When counseling family members and/or surrogates of patients with msTBI admitted to an ICU, should the CRASH-basic and CRASH-CT models be considered reliable predictors of poor functional outcome at 6 months and beyond?”

Description of the predictor: See PICOT Question 21.

Recommendation: When counseling family members and/or surrogates of patients with msTBI admitted to an ICU, we suggest that the CRASH-basic and CRASH-CT clinical prediction models be considered moderately reliable predictors of poor functional outcome at 6 months and beyond (weak recommendation; moderate-quality evidence).

Rationale: The body of evidence was downgraded for risk of bias. Among the studies of the CRASH-basic model, one [45] had a relatively small sample size, limited information on the analysis, a small number of patients with the outcome of interest, and limited information on how outcome was measured. Two of four studies did not report calibration [45, 54]. All studies did not address the impact of WLST and were therefore at risk of bias from the self-fulfilling prophecy. However, two large multicenter studies [43, 44] and one medium-size study [54] were judged to have an overall low risk of bias. For the CRASH-CT model, three studies were included, two of which one had an overall low risk of bias [43, 46], but the other [54] had a high risk of bias due to lack of calibration, minimal information on the analysis, and risk for self-fulfilling prophecy.

#### PICOT Question 29

“When counseling family members and/or surrogates of patients with msTBI admitted to an ICU, should the IMPACT-core and IMPACT-extended (core + CT) models be considered reliable predictors of poor functional outcome at 6 months and beyond?”

Description of the predictor: See PICOT Question 22.

Recommendation: When counseling family members and/or surrogates of patients with msTBI admitted to an ICU, we suggest that the IMPACT-core and IMPACT-extended (core + CT) clinical prediction models be considered moderately reliable predictors of poor functional outcome at 6 months and beyond (weak recommendation; moderate-quality evidence).

Rationale: The body of evidence was downgraded for risk of bias. For the IMPACT-core model, three of eight studies had potential bias from small sample size [45], restriction of eligibility to age 65 and older [50], a third of patients having mild TBI [47], limited reporting of outcome assessment [45, 50], insufficient data on how the analysis was performed, and lack of calibration [45, 54]. In one study [48], there was variability in the time point of outcome assessment (between 6 and 12 months). However, several large studies had low risk of bias [23, 44, 46]. For the IMPACT-extended model, one study restricted eligibility to age 65 and older and had insufficient detail on analysis [50]. Another study did not report calibration [54]. In one study [48], there was variability in the time point of outcome assessment (between 6 and 12 months). The remaining studies had low risk of bias [23, 44, 46]. All studies did not address the impact of WLST and were therefore at risk of bias from the self-fulfilling prophecy.

#### PICOT Question 30

“When counseling family members and/or surrogates of patients with msTBI admitted to an ICU, should the IMPACT core + CT + lab model be considered a reliable predictor of poor functional outcome at 6 months and beyond?”

Description of the predictor: See PICOT Question 23.

Recommendation: When counseling family members and/or surrogates of patients with msTBI admitted to an ICU, we suggest that the IMPACT core + CT + lab clinical prediction model be considered a moderately reliable predictor of poor functional outcome at 6 months and beyond (weak recommendation; moderate-quality evidence).

Rationale: The body of evidence was downgraded for risk of bias. One study restricted eligibility to age 65 and older and included limited information on outcome assessment [50]. In one study [48], there was variability in the time point of outcome assessment (between 6 and 12 months). The other two studies had low risk of bias [23, 46]. All studies did not address the impact of WLST and were therefore at risk of bias from the self-fulfilling prophecy.

#### PICOT Question 31

“When counseling family members and/or surrogates of patients with msTBI admitted to an ICU, should the Marshall CT classification be considered a reliable predictor of poor functional outcome at 6 months and beyond?”

Description of the predictor: See PICOT Question 24.

Recommendation: When counseling family members and/or surrogates of patients with msTBI admitted to an ICU, we suggest that the Marshall CT classification alone not be considered a reliable predictor of poor functional outcome at 6 months and beyond (weak recommendation; low-quality evidence).

Rationale: The body of evidence was downgraded for risk of bias from study participation, applicability, and analysis (missing data excluded without imputation) [47, 55]. Studies did not address the impact of WLST and were therefore at risk of bias from the self-fulfilling prophecy [47, 52, 55]. Indirectness was present, as a quarter [52] to 40% of patients included had only mild TBI [47, 55]. The original Marshall publication [51] did not meet our inclusion criteria, as it did not report model discrimination.

#### PICOT Question 32

“When counseling family members and/or surrogates of patients with msTBI admitted to an ICU, should the Rotterdam CT model be considered a reliable predictor of poor functional outcome at 6 months and beyond?”

Description of the predictor: See PICOT Question 25.

Recommendation: When counseling family members and/or surrogates of patients with msTBI admitted to an ICU, we suggest that the Rotterdam CT model alone not be considered a reliable predictor of poor functional outcome at 6 months and beyond (weak recommendation; low-quality evidence).

Rationale: The body of evidence was downgraded for risk of bias from study participation, applicability, and analysis (missing data excluded without imputation) [47, 55]. Indirectness was present, as a quarter [52] to 40% of patients included had only mild TBI [47, 55]. Studies did not address the impact of WLST and were therefore at risk of bias from the self-fulfilling prophecy [47, 55].

#### PICOT Question 33

“When counseling family members and/or surrogates of patients with msTBI admitted to an ICU, should the Helsinki CT model be considered a reliable predictor of poor outcome at 6 months and beyond?”

Description of the predictor: See PICOT Question 26.

Recommendation: When counseling family members and/or surrogates of patients with msTBI admitted to an ICU, we suggest that the Helsinki CT model alone not be considered a reliable predictor of poor outcome at 6 months (strong recommendation; low-quality evidence).

Rationale: The body of evidence was downgraded for risk of bias from study participation, applicability, and analysis (missing data excluded without imputation) [47, 55]. Indirectness was present, as a quarter to 40% of patients included had only mild TBI [47, 55]. Studies did not address the impact of WLST and were therefore at risk of bias from the self-fulfilling prophecy [47, 52, 55].

#### PICOT Question 34

“When counseling family members and/or surrogates of patients with msTBI admitted to an ICU, should the Stockholm CT model be considered a reliable predictor of poor functional outcome at 6 months and beyond?”

Description of the predictor: See PICOT Question 27.

Recommendation: When counseling family members and/or surrogates of patients with msTBI admitted to an ICU, we suggest that the Stockholm CT model alone not be considered a reliable predictor of poor functional outcome at 6 months and beyond (weak recommendation; low-quality evidence).

Rationale: The body of evidence was downgraded for risk of bias in the domains of applicability, outcome assessment (performed at variable time points), and analysis (calibration not reported). Studies did not address the impact of WLST and were therefore at risk of bias from the self-fulfilling prophecy [52, 55]. Indirectness was present, as a quarter to 40% of patients included had only mild TBI.

## Future Directions

A broad body of literature has analyzed the association of clinical, radiological, and laboratory features with neurological outcome after msTBI. Several findings on admission and during therapy have been shown to have an association with poor outcome or mortality, but the risk of bias in the published studies is too high for the panel to recommend their use in isolation when counseling surrogates and family members, particularly in the context of limitations of therapy and WLST. Some existing prediction models, above all the CRASH and IMPACT models, show moderate reliability and may be used with caution and appropriate acknowledgment of uncertainty when prognosticating outcome after msTBI.

Based on the most common study limitations identified in our systematic review, future studies should consider the following general principles to be considered for recommendation for neuroprognostication:

1. Future studies should perform multivariate analysis that includes most widely accepted individual factors, at least age, and GCS score. They should also mitigate the risk of self-fulfilling prophecies through blinding of clinicians to predictors not integral to clinical care and, where feasible, restrictions on early WLST.
2. Because no individual prognostic variable can be expected to be strong enough to decide on the direction of therapy alone, possible predictors should be assessed as an add-on to validated prognostication models or in isolation. To be relevant for neuroprognostication, they must improve the discrimination and calibration of these prognostication models and have low risk of bias in the domains of participation, outcome determination, analysis, and self-fulfilling prophecy.
3. Mortality and long-term functional outcome of patients with msTBI are not merely defined by admission variables. Therefore, inclusion of subsequent clinical occurrences, such as ICU complications, worsening of radiographic features and brain edema, and more, with appropriate use of statistical modeling (e.g., survival analysis with inclusion of time-to-event or Bayesian modeling), is necessary to move the field of neuroprognostication forward. This would add information not only to which variables may help improve prognostication but also to when the right time point is for more accurate prognostication.
4. Finally, the majority of included studies for both individual variables and prediction models assessed functional outcome only up until 6 months. More recently, several clinical trials have documented that recovery from msTBI continues beyond 6 months, suggesting that an end point of 6 months may be too early to determine recovery [56–58].

## Conclusions

These guidelines provide recommendations on the use of predictors of mortality and functional outcome in msTBI in the context of counseling surrogates and family members and suggest broad principles of neuroprognostication.

### Supplementary Information

Below is the link to the electronic supplementary material.

Supplementary file1 (DOCX 16 KB)

Supplementary file2 (XLSX 255 KB)

Supplementary file3 (XLSX 143 KB)
