# Supplementary material for: Guidelines for Neuroprognostication in Critically Ill Adults with Moderate–Severe Traumatic Brain Injury
Source: Neurocrit Care. 2024 Feb 17;40(2):448–76. doi: 10.1007/s12028-023-01902-2 (PMC10959796; doi:10.1007/s12028-023-01902-2)
Supplement: Supplementary file 1 — Supplementary file1 (DOCX 16 KB) [file 12028_2023_1902_MOESM1_ESM.docx]

**Supplementary Appendix 1**

**Librarian search string**

Database: All Ovid Medline <1946 - present>

Search Strategy:

--------------------------------------------------------------------------------

1 exp Brain Injuries, Traumatic/ (10634)

2 exp "wounds and injuries"/ (856274)

3 exp Craniocerebral Trauma/ (149480)

4 (trauma* or traumatic*).ti,ab. (330955)

5 exp Wounds, Gunshot/ (14717)

6 exp Wounds, Penetrating/ (35005)

7 Head Injuries, Penetrating/ (935)

8 or/2-7 (1025361)

9 exp brain injuries/ (63641)

10 encephalopath*.ti,ab. (44890)

11 brain injur*.ti,ab. (58195)

12 TBI.ti,ab,kf. (21626)

13 exp Intracranial Hemorrhages/ (67011)

14 (Traumatic adj5 subarachnoid).ti,ab. (882)

15 (Traumatic adj5 subdural).ti,ab. (666)

16 (contusion adj5 (brain or trauma*)).tw. (1321)

17 (diffuse axonal adj5 (brain or trauma)).tw. (223)

18 exp brain/ (1147709)

19 9 or 10 or 11 or 12 or 13 or 14 or 15 or 18 (1297310)

20 8 and 19 (106418)

21 1 or 20 (106418)

22 exp Analysis of Variance/ [includes Multivariate Analysis] (326771)

23 Multivariate.tw. (293720)

24 treatment outcome/ (885811)

25 outcome.tw. (887975)

26 mortality/ or mo.fs. (563611)

27 "Predictive Value of Tests"/ (188131)

28 Disease Progression/ (146077)

29 prediction.tw. (213326)

30 prognostic*.tw. (268771)

31 prognos*.tw. (536821)

32 exp Quality of Life/ (171899)

33 "Quality of life".tw. (241815)

34 scale.ti,ab. (629667)

35 score.ti,ab. (478944)

36 scoring tool*.tw. (605)

37 disability evaluation/ (44760)

38 Survival/ (4559)

39 mortality/ or mo.fs. or death/ (579364)

40 Time factors/ (1143362)

41 22 or 24 or 25 or 26 or 27 or 29 or 30 or 31 or 32 or 34 or 35 or 36 or 37 or 38 or 40

(4506153)

42 21 and 41 (34556)

43 prognosis.sh. or diagnosed.tw. or cohort:.mp. or predictor:.tw. or death.tw. or exp models,

statistical/ [validated hedge from the Health Information Research Unit, McMaster University]

(2446889)

44 21 and 43 (19261)

45 42 or 44 (42555)

46 exp cohort studies/ [includes: follow-up studies/, longitudinal studies/, prospective studies/,

retrospective studies/, controlled before-after studies/, cross-sectional studies/, or historically

controlled study/] (1824723)

47 (Follow-up or longitudinal or prospective or retrospective or before-after or cross-sectional

or controlled).tw. (2597128)

48 (predict* or predictor or prognos* or prognost*).ti. (415631)

49 or/46-48 (3640205)

50 45 and 49 (17323)

51 animals/ not (humans/ and animals/) (4515460)

52 limit 50 to ("newborn infant (birth to 1 month)" or "infant (1 to 23 months)" or "preschool child

(2 to 5 years)" or "child (6 to 12 years)") (4102)

53 letter.pt. (1015878)

54 limit 50 to (address or autobiography or bibliography or biography or case reports) (831)

55 51 or 52 or 53 or 54 (5501065)

56 50 not 55 (11600)
